# Socio-economic Correlates and Spatial Heterogeneity in the Prevalence of Asthma among Young Women in India

**DOI:** 10.1186/s12890-020-1124-z

**Published:** 2020-07-14

**Authors:** Shri Kant Singh, Jitendra Gupta, Himani Sharma, Sarang P. Pedgaonkar, Nidhi Gupta

**Affiliations:** 1 Department of Mathematical Demography & Statistics, International Institute for Population Sciences, Govandi Station Road, Deonar, Mumbai, 400088 India; 2 Department of Population Policies & Programmes, International Institute for Population Sciences, Govandi Station Road, Deonar, Mumbai, 400088 India; 3 International Institute for Population Sciences, Govandi Station Road, Deonar, Mumbai, 400088 India

**Keywords:** Asthma, Environmental & Ecological Factors, Tobacco use, Lifestyle, Moran’s I, Spatial autocorrelation, and autoregression

## Abstract

**Background:**

Asthma is one of the leading causes of disease burden when measured in terms of disability adjusted life years, despite low prevalence of self-reported cases among young women. This paper deals with the meso-scale correlates and spatial heterogeneity in the prevalence of self-reported Asthma across 640 districts in India, using a nationally representative sample of 699,686 women aged 15–49 years from all 36 States/UTs under NFHS-4 (2015–16).

**Methods:**

Analytical methods used in this paper include multivariate logistic regression to examine the adjusted effects of various independent variables on self-reported Asthma and poor-rich ratios (PRR) and concentration index (CI) to understand the economic inequalities in the prevalence of Asthma. For the spatial analysis in the prevalence of Asthma, univariate and bivariate local Moran’s I statistic have been computed in addition to measure of spatial autocorrelation and auto regression using spatial error and spatial lag models.

**Results:**

Results highlight that women’s education was an important marker to the prevalence of Asthma. Smoking tobacco in any form among women were significantly more likely to suffer from Asthma. The prevalence of Asthma was further aggravated among women from the households without a separate room for kitchen, as well as those using unclean fuel for cooking. The poor-rich ratio in the prevalence of Asthma across various States/UTs in India depict inherent inequality. An analysis of spatial clustering in the prevalence of Asthma based on spatial autocorrelation portrays that Moran’s I values were significant for improved source of drinking water, clean fuel used for cooking, and household environment. When spatial weights are taken into consideration, the autoregression model noticeably becomes stronger in predicting the prevalence of Asthma.

**Conclusions:**

Any programmatic effort to curb the prevalence of Asthma through vertical interventions may hinge around the use of clean fuel, poverty, and lifestyle of subjects, irrespective of urban-rural place of their residence, environmental and ecological factors.

## Background

Asthma is a chronic disease characterized by recurrent attacks of breathlessness and wheezing, which vary in severity from person to person [1]. It results from the chronic inflammation of the air passages in the lungs and affects the sensitivity of the nerve endings in the airways, making them easily irritable and hyper-responsive to exposure of small quantity of allergens or irritants. During an Asthma attack, the airways constrict due to swollen bronchial tube lining and spasm of the muscles of the airways, reducing the flow of air into and out of the lungs. The obstruction of airways in Asthma is recurrent and reversible. The symptoms of Asthma also include cough, chest tightness, fatigue, agitation, chest or abdominal pain and increased pulse rate. Symptoms can reoccur multiple times and may become worse during physical activity or at night. Recurrent Asthma symptoms frequently cause sleeplessness, daytime fatigue, reduced activity levels, and school and work absenteeism [2]. Asthma is a complex disease that impairs the social, physical, and psychological well-being of the affected. According to the GBD 2015, Asthma ranked among the top 20 conditions causing disability globally and ranked 23^rd^ as causes of disease burden when measured by disability-adjusted life years [3].

Asthma is prevalent in all countries and in all age-groups however; it is the most common chronic disease among children. Although Asthma cannot be cured, appropriate management can control the disease and enable people to enjoy a good quality of life. It is often under-diagnosed and under-treated, creating a substantial burden on individuals and families and possibly restricting individuals’ activities for a lifetime. A majority (over 80%) of Asthma deaths occur in low- and lower-middle income countries [4]. According to WHO 2016, Asthma affects 235 million people worldwide, out of which 15–20 million people are from India [4]. In India, the prevalence of self-reported Asthma is 2.00% among women aged 15–49 years and 1.00% among young women aged 15–19 years as well as men aged 15–49 years as per the latest report [5]. The Asthma related conditions seem to be under-reported primarily due to underestimation of the signs and symptoms of the disease, and considering it as a normal phenomenon. Further, the patients often experience symptoms but they hesitate to present these symptoms to a physician probably because they refuse to take anti-asthmatic drugs, this augments the burden laid on to a general practitioner, leading to late interventions due to patients’ perception and values [6]. Several evidences from scientific literature have been found about the high prevalence of underdiagnoses and over diagnosis of Asthma. The undiagnosed Asthma is related to poorer health-related quality of life and more absenteeism from work and school while over diagnosed Asthma is connected with unnecessary use of medications, the burden of increased drug costs, and the lost opportunity to diagnose the true cause of their respiratory symptoms [7].

On exploring the literature to understand the environmental risk factors for Asthma, it was observed that exposure to indoor pollution caused due to the use of biomass fuels by the poor in low and middle-income countries like India, is a major risk factor, which has a significant impact upon the health of the communities. The causes of Asthma are yet to be fully understood, however, dominant risk factors for the development of Asthma are a complex interplay of genetic susceptibility and exposure to environmental particulate allergens and substances like indoor and outdoor allergens, tobacco smoke, chemical irritants in the workplace and air pollution. Asthma attacks can also be triggered by exposure to cold air, extreme emotional states such as anger or fear, physical exercises, and certain drugs like aspirin, other non-steroid anti-inflammatory drugs, and beta-blockers [4]. A study by Smith, (2000) in India reflects that though biomass fuels, such as dung-briquette and firewood are cheaper than the modern fuels, the indoor pollution caused from burning biomass fuels incurs high health costs [8]. A strong association between cooking smoke and the prevalence of Asthma was observed. Furthermore, an even stronger association was observed for older women, given the fact that they are relatively more exposed to cooking fuel than males over their life course [9]. Gender differences in the prevalence of respiratory disorders like Asthma have been reported in the studies undertaken in rural areas of Uttar Pradesh, Rajasthan and Himachal Pradesh with women at greater risk due to their exposure to indoor pollution [10].

Third NHANES as well as ECRHS highlighted the causal relation between environmental tobacco smoke and chronic respiratory problems. It concludes that consumption and exposure to environmental tobacco smoke leads to lung and respiratory deficits, especially among adult females [11, 12]. A study by Eisner, (2002) showed the relationship between Asthma and Airborne moulds; suggesting that sensitization to moulds might be involved in the severity of Asthma [13]. Asthma contributes significantly to the total burden of deaths or DALYs [14]. India, comprising of 36 States/UTs, further bifurcated into 640 districts, has profound differences in socio-cultural, behavioural and climatic conditions, which bears almost one-tenth of the global burden of Asthma. However, there is no systematic effort to look at the spatial variation in the prevalence of Asthma across 640 districts of India, in spite of the several rounds of large-scale national level surveys having been undertaken across the country until date. Moreover, women are at higher risk of developing the condition, as reflected in the various studies undertaken in both developed and developing countries [8–10]. This study attempts to answer following research questions. Firstly, it examines whether there exists a pattern in the spatial clustering in the prevalence of Asthma among women across 640 districts in the country. Secondly, it looks into the socio-economic and behavioural factors affecting the prevalence of Asthma among women in India. In view of these research questions, the major objectives of the paper are to understand the key socio-economic, behavioural and ecological determinants of Asthma among women aged 15–49 years in India and the spatial variations in its clustering.

## Methods

The data used in this study have been taken from the National Family Health Survey (NFHS)- 4 conducted in 640 districts of India in 2015–2016. NFHS surveys is conducted under the stewardship of the Ministry of Health and Family Welfare (MoHFW), Government of India and the International Institute for Population Sciences (IIPS), Mumbai, has been the nodal agency. The survey provides information on demographic and health indicators at the national, regional, and state level and from 2015 onwards, it provides information at the district level, too. NFHS-4 collected information from a nationally representative sample of 601,509 households, 699,686 women aged 15–49 years from the aforementioned households [5]. It is worthwhile to mention that NFHS-4 has adopted a modular approach, but the information regarding Asthma was collected from both men and women. The information obtained from women has been analyzed in the present study. The information on the emerging health issues for each of 640 districts in the country are available in public domain for use [15].

### Dependent variable

The outcome variable for the study is Asthma among women aged 15–49 years. In the survey, Asthma is a self-reported case as all the interviewed women were asked whether they have Asthma and if yes, whether they have sought any treatment or not. Among 699,686 women included in the study, around 11,194 women reported suffering from Asthma at the time of the survey, which is compiled into the available data source along with their socio-economic, behavioural, environmental, and ecological characteristics. The participation of the Indian women in the survey was 98%.

### Independent variables

A set of important determinants of Asthma have been considered for the study. The determinants were selected based on existing literature and data availability. The determinants of household environment included improved source of drinking water, improved source toilet facility, clean fuel for cooking food, a separate room used as the kitchen, household with no crowding, consumption of tobacco in any form. In addition to this, some socio-economic determinants, i.e. education of women, type of residence, caste/tribe, religion, national regions, wealth quintiles, tobacco use and exposure to mass media have been used in the study. The correlation test between confounders was performed to check highly correlated variables.

### Statistical measures

Logistic regression analysis has been applied to examine the adjusted effects of various independent variables on self-reported Asthma among women aged 15–49 years. The logistic regression model is commonly estimate by maximum likelihood function. For each of those above mentioned outcome variables, logistic model takes the following general form;

$$ \mathrm{Logit}\ \mathrm{P}=\mathrm{Ln}\ \left[\mathrm{P}/1\hbox{-} \mathrm{P}\right]={b}_0+{b}_1+{b}_1{x}_1+{b}_2{x}_2+\hbox{-} \hbox{-} \hbox{-} \hbox{-} \hbox{-} \hbox{-} \hbox{-} \hbox{-} \hbox{-} \hbox{-} \hbox{-} \hbox{-} +{b}_1{x}_i+{e}_i $$

**Table 1:** Descriptive statistics of household environment and background characteristics among Asthmatic young women, India 2015–16

Household, Env. & Background Characteristics	Percent	N
**Improved source of drinking water**		
No	15.8	1771
Yes	84.2	9423
**Improved source toilet facility**		
No	35.5	3978
Yes	64.5	7216
**Clean cooking fuel**		
No	54.4	6087
Yes	45.6	5107
**Separate room used as kitchen**		
No	49.7	5559
Yes	50.3	5635
**Household with no crowding**		
No	52.7	5897
Yes	47.3	5297
**Consumption of any tobacco**		
No	90.8	10,160
Yes	9.2	1034
**Level of Education**		
No education	31.7	3552
Primary	15.6	1749
Secondary	43.6	4881
Higher	9.0	1012
**Place of Residence**		
Urban	36.7	4108
Rural	63.3	7086
**Caste**		
SC	19.2	2148
ST	8.4	939
OBC	43.1	4824
Others	29.3	3282
**Religion**		
Hindu	81.2	9086
Muslim	12.4	1392
Others	6.4	716
**Region**		
Eastern	26.5	2961
Western	16.1	1801
Northern	14.4	1615
Southern	34.6	3867
North-eastern	2.5	278
Central	6.0	671
**Wealth Index**		
Poorest	15.8	1773
Poorer	19.1	2139
Middle	21.2	2371
Richer	23.6	2638
Richest	20.3	2272
**Mass media**		
No	16.5	1849
Yes	83.5	9345
**India**		**11,194**

Where b_0_, b_1_, b_2_, … … b_i_ represents the co-efficient of each of the predictors included in the model, while e_i_ is an error term.

Poor-rich ratios (PRR) and concentration index (CI) have been used to understand the economic inequalities in the prevalence of Asthma among women aged 15–49 years in India. The concentration curves and concentration indices are used to measure the overall inequalities in the particular health outcome among the wealth quintiles [16]. The CI is a measure of inequality and is defined as twice the area between the concentration curve and the diagonal, and it varies between − 1 and + 1 [17]. It denotes that if the line of concentration curve lies above the equality line, depicting the disproportionate distribution of a particular health burden among more deprived groups, values lie between 0 and − 1. Therefore, if the values are going towards − 1, it indicates a greater inequality in this study. The spatial analysis, mainly two software’s, i.e., ArcGIS and GeoDa have been used in the study. It is a generalization of Pearson’s correlation coefficient. Spatial autocorrelation indicates the degree to which data points are similar or dissimilar to their spatial neighbours by Moran’s I value [18]. Univariate local indicators of spatial association (LISA) measure the correlation of neighbourhood values around a specific spatial location [19].

## Results

The results from the recently completed NFHS-4 data portray that 1.94% of the women aged 15–49 years in India were suffering from Asthma. Table 1 presents the descriptive statistics of household environment and background characteristics while while replace by among Asthmatic young women in India during 2015-16. Table 2 presents the adjusted and unadjusted prevalence of Asthma among women in India by some selected background and behavioural characteristics. It is evident that women who consume tobacco in any form are more likely to suffer from Asthma (3.35%). The results remain unchanged even after adjusting the prevalence of Asthma for some other predictors included in the model. Logistic regression odds ratios (OR) revealed that women who consumed tobacco were significantly more likely to have Asthma (OR = 1.66, *p* < 0.01).

**Table 2:** Prevalence and Adjusted effect of Asthma among young women aged 15–49 years by household environment and background characteristics, India 2015–16

Household, Env. & Background Characteristics	Percent	Odds Ratio	Lower	Upper
**Improved source of drinking water**				
No®	2.16			
Yes	1.90	1.00	0.95	1.05
**Improved source toilet facility**				
No®	1.81			
Yes	2.02	1.07**	1.01	1.13
**Clean cooking fuel**				
No®	1.84			
Yes	2.07	1.03	0.97	1.09
**Separate room used as kitchen**				
No®	1.95			
Yes	1.93	0.95**	0.91	0.99
**Household with no crowding**				
No®	1.81			
Yes	2.11	1.11***	1.07	1.15
**Consumption of any tobacco**				
No®	1.86			
Yes	3.35	1.66***	1.56	1.76
**Level of education**				
No education®	2.24			
Primary	2.43	0.93**	0.88	0.99
Secondary	1.79	0.62***	0.59	0.65
Higher	1.37	0.44***	0.40	0.48
**Place of residence**				
Urban®	2.05			
Rural	1.88	1.02	0.97	1.07
**Caste**				
SC®	1.82			
ST	1.77	0.92**	0.85	0.98
OBC	1.92	1.02	0.97	1.08
Others	2.10	1.22***	1.15	1.30
**Religion**				
Hindu®	1.95			
Muslim	1.75	0.84***	0.79	0.89
Others	2.20	1.18***	1.10	1.27
**Region**				
Eastern®	2.32			
Western	1.42	0.64***	0.60	0.69
Northern	1.20	0.59***	0.56	0.63
Southern	2.94	1.48***	1.39	1.58
North-eastern	1.37	0.68***	0.62	0.73
Central	1.86	0.94	0.87	1.01
**Wealth index**				
Poorest®	1.73			
Poorer	1.89	1.14***	1.06	1.21
Middle	2.00	1.11***	1.03	1.20
Richer	2.16	1.25***	1.14	1.37
Richest	1.87	1.40***	1.26	1.56
**Mass media**				
No®	1.70			
Yes	1.99	1.18***	1.12	1.25
**India**	**1.94**			

The values of the odds ratio are significant at *p < 0.10, **p < 0.05, ***p < 0.01with respect to®reference category

The prevalence of Asthma is found to be higher among women from the households who did not use a separate room as a kitchen (1.95%). Supporting the statement, the odds ratio also showed that women who use a separate room as kitchen were comparatively less likely to suffer from Asthma (OR = 0.95, *p* < 0.05) than those who did not use a separate room as kitchen. Women’s education has been an important marker to the attitude, perceptions, and behaviour towards their health and wellbeing. Results portrayed that the prevalence of Asthma among women was negatively associated with their educational attainment, i.e. the higher the level of education of women, lower was the self-reported prevalence of Asthma. The odds of the prevalence of Asthma were significantly lower among women who have completed primary (OR = 0.93, *p* < 0.05) secondary (OR = 0.62, *p* < 0.01) and higher secondary (OR = 0.44, *p* < 0.01) schooling as compared to the women who did not have any schooling.

The prevalence of Asthma is found to be higher among women from household with no crowding in their households and hailing from households belonging to higher wealth quintiles. The women from household with no crowding were 11.00% more likely to suffer from Asthma (OR = 1.11, *p* < 0.01). Similarly, women belonging to the richest wealth quintiles were 40.00% more likely to suffer from Asthma (odds ratios for women from different wealth quintiles ranges from 1.11 to 1.40, *p* < 0.01). A similar contrasting pattern in the prevalence of Asthma was found by their exposure to media, where those who reported having regular media exposure were significantly more likely to suffer from Asthma (OR = 1.18, *p* < 0.01). A similar pattern in the prevalence of Asthma was observed even among women from non-SC/ST and non-OBC households and religions other than Hinduism and Islam. As the prevalence of Asthma has been recorded based on reporting of women at the time of survey rather than using any standard tests. It has been the case in other bio-markers, better reporting among the people who belong to socio-economically better-off households and are better aware of signs and symptoms of the disease could be one of the possible reasons behind this unexpected pattern.

**Map 1:**
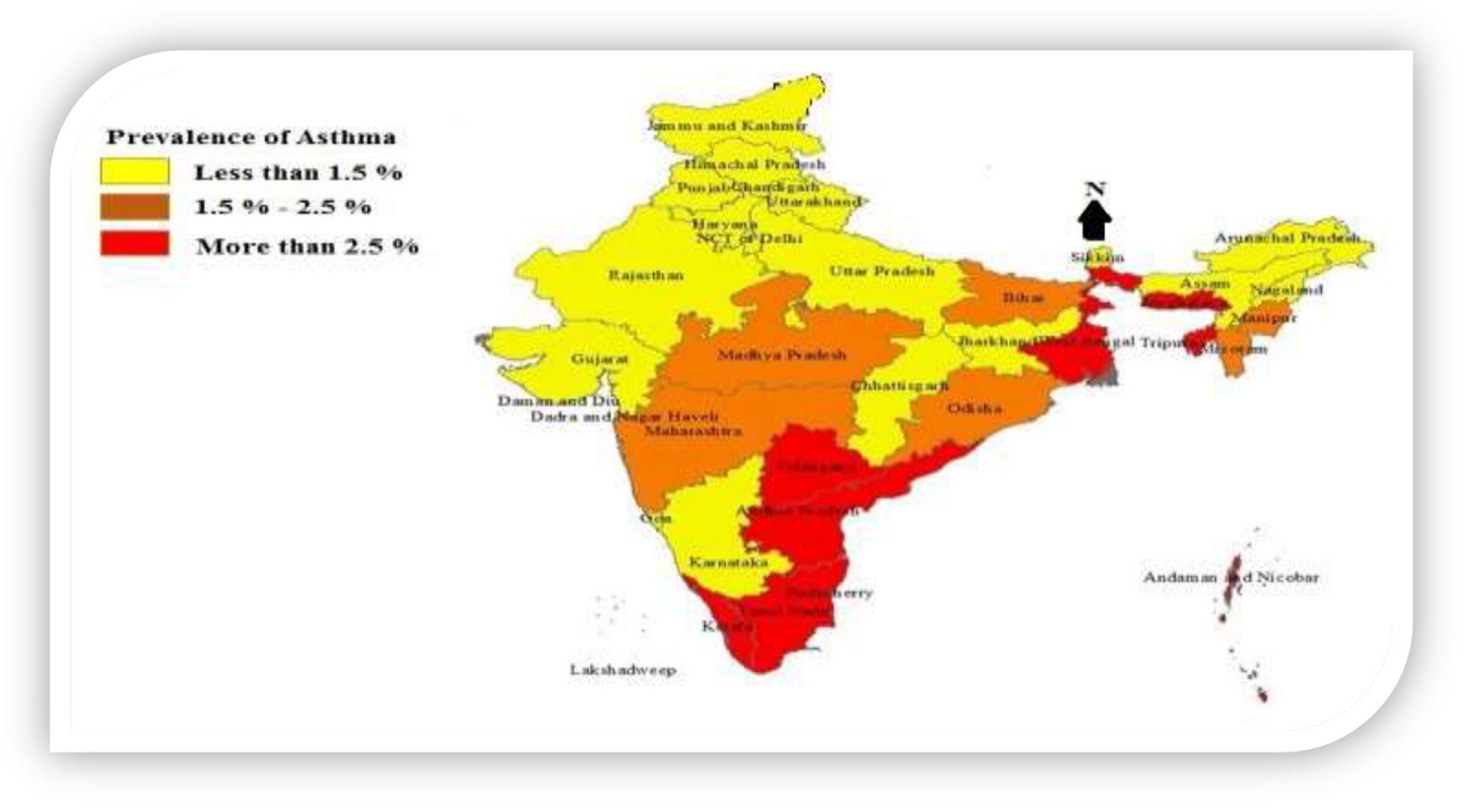
Prevalence of Asthma by different State/UTs of India, 2015–16. Note: These maps are only indicative and do not portray the political/administrative boundaries of India. Some of the districts of Jammu and Kashmir have no data coverage [20]

**Map 2:**
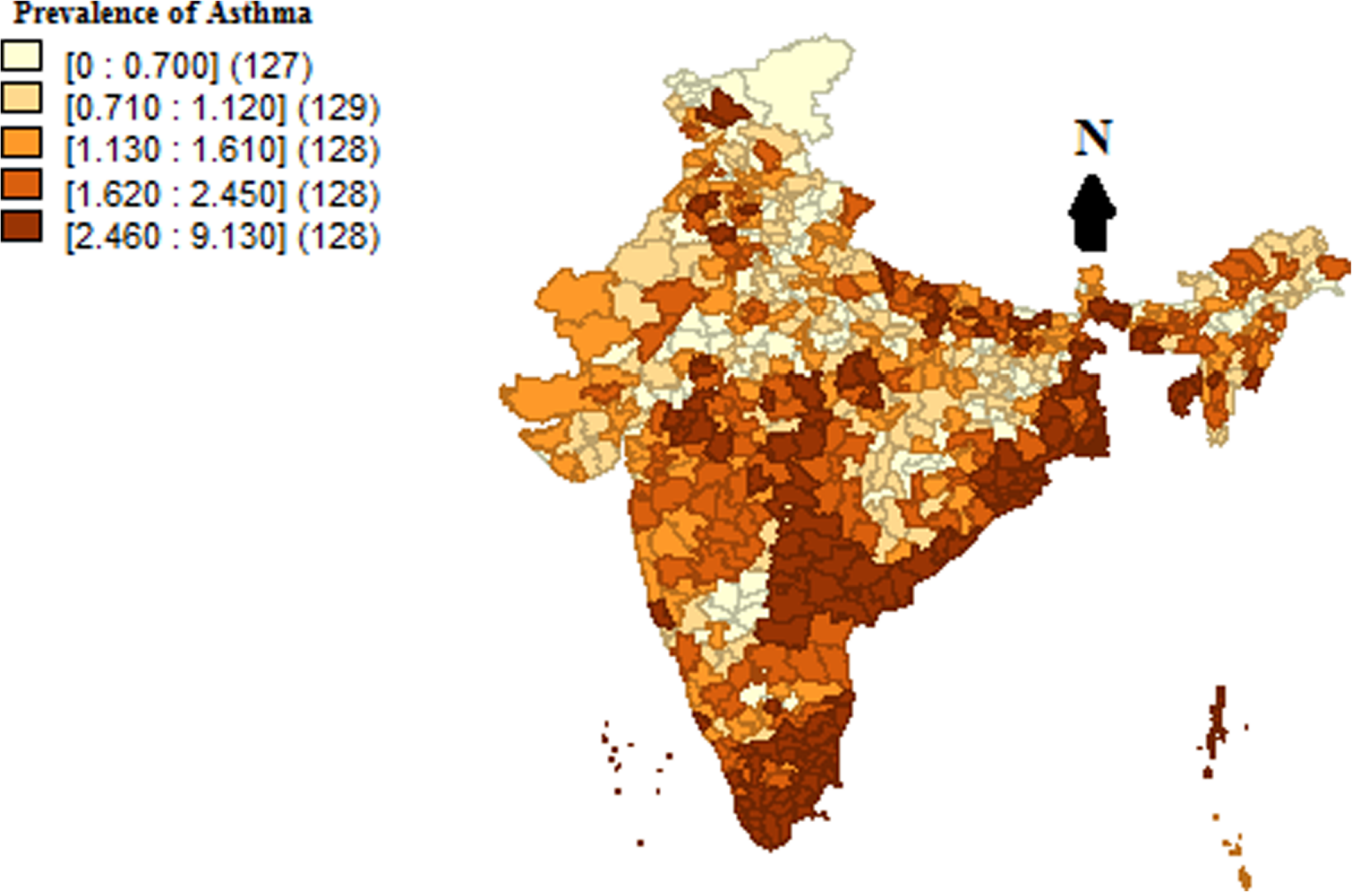
Prevalence of Asthma by different districts of India, 2015–16. Note: These maps are only indicative and do not portray the political/administrative boundaries of India. Some of the districts of Jammu and Kashmir have no data coverage [20]

The prevalence of Asthma among women was also varying by environmental and ecological variations existing across UTs in India have been presented in Table/regions of the country. It is evident from Map 1 that the prevalence of Asthma among women was the lowest in the northern region (1.20%) including states of north-east regions of the country, while there were relatively larger prevalence in the southern region namely Kerala, Tamil Nadu, Andhra Pradesh, Telangana, and some of the states in the eastern and north-eastern regions namely West Bengal, Assam, Tripura, Sikkim etc.. These findings are also supported with the results presented in Table 2, where the odds ratio of Asthma is significantly lower among women belonging to the northern region (OR = 0.59, *p* < 0.01) as compared to their eastern counterpart. Map 2 portrays the prevalence of Asthma in 640 districts of India, amongst which 128 districts had a higher prevalence of Asthma, which is represented by the darkest shade of brown in the map. Women from the districts mostly belonging to the eastern coastal regions of the country, including some districts of the central region, were reported suffering from Asthma at the time of survey. It is evident from the map that higher concentration among women from completely eastern coastal line is suffering from Asthma. Most of the districts of Andhra Pradesh, Telangana and Tamil Nadu including some districts of Madhya Pradesh were found to have a higher prevalence of Asthma.

The economic inequalities in the prevalence of Asthma among women aged 15–49 years across the UTs in India have been presented in Table/UTs in India have been presented in Table 3. The overall poor-rich ratio among women having Asthma was 0.93, which indicates that wealth wise prevalence of Asthma does not portray a variation; but there is a tremendous variability across States/ UTs. It is found that 192 districts (30.0%) were having the prevalence of Asthma much above the national average (1.94%). It is clearly visible from the results presented in the table that women belonging to richest wealth quintile were more likely to suffer from Asthma than those from the poorest wealth quintiles (PRR = 0.93). However, the state-specific variation in the poor-rich ration is the real issue of concern for any health interventions to address the prevalence of Asthma and bronchitis in the country. The poor-rich ratios vary from 0.00 in the State/UTs of Goa, Sikkim, Chandigarh, Daman and Diu, Delhi, Lakshadweep and Puducherry to 3.50 in Kerala. The poor-rich ratios are found higher in the states viz. Kerala (3.50), Himachal Pradesh (3.40), Bihar (2.30), and Punjab (2.20). The prevalence of Asthma among women is observed increasing trend with wealth quintiles in Lakshadweep (0.00–4.50) and Meghalaya (1.70–4.10). The values of CI for Asthma ranges from − 0.12 (Jammu and Kashmir) to 0.30 (Lakshadweep) suggesting that Asthma increases with increasing economic inequalities, thereby being disadvantageous to the richer. There is a larger concentration of Asthma among women belonging to Lakshadweep (0.30), Karnataka (0.18), Odisha (0.13), Dadra and Nagar Havel (0.12), Chhattisgarh (0.11) and Meghalaya (0.10) etc.. The economically progressive states in southern and eastern parts of India recorded a relatively higher prevalence of Asthma.

**Table 3:** Comparison of prevalence of Asthma among young women of aged 15–49 years within different level of wealth quintiles, poor-rich ratio (PRR), Wagstaff concentration index (CI) and standard error (SE) by State/UTs of India, 2015–16

State/UTs	Poorest	Poorer	Middle	Richer	Richest	PRR	CI	SE
Andaman and Nicobar Is	5.9	4.2	4.0	4.3	5.1	1.15	0.02	0.06
Andhra Pradesh	2.7	2.6	2.6	3.2	3.6	0.76	0.05	0.04
Arunachal Pradesh	1.2	0.9	1.0	1.7	1.2	1.05	0.07	0.05
Assam	0.9	0.9	1.0	0.8	1.7	0.52	0.07	0.04
Bihar	1.9	1.9	1.9	1.3	0.8	2.34	−0.06	0.02
Chandigarh	0.0	6.8	0.0	1.2	1.3	0.00	−0.06	0.18
Chhattisgarh	0.7	0.6	1.0	1.1	1.3	0.53	0.11	0.04
Dadra and Nagar Havel	2.7	0.0	0.0	1.7	3.5	0.76	0.12	0.22
Daman and Diu	0.0	0.4	2.1	0.5	0.8	0.00	0.06	0.19
Goa	0.0	0.0	1.0	1.8	0.7	0.00	−0.11	0.11
Gujarat	1.7	1.6	1.2	1.4	1.2	1.49	−0.07	0.04
Haryana	1.4	1.5	1.7	1.4	1.2	1.17	−0.05	0.04
Himachal Pradesh	4.0	0.2	1.0	1.0	1.2	3.40	0.06	0.06
Jammu and Kashmir	1.4	0.9	0.8	0.9	0.8	1.65	−0.12	0.04
Jharkhand	0.6	0.7	0.8	0.7	0.7	0.87	0.04	0.04
Karnataka	0.7	0.9	0.9	1.7	2.8	0.26	0.18	0.04
Kerala	10.9	1.7	3.5	3.1	3.1	3.48	0.01	0.03
Lakshadweep	0.0	0.0	1.2	2.3	4.5	0.00	0.30	0.11
Madhya Pradesh	1.7	1.9	1.8	2.1	1.9	0.93	0.01	0.02
Maharashtra	2.3	2.0	1.8	2.0	1.5	1.52	0.01	0.03
Manipur	1.7	1.7	1.6	1.4	1.7	0.99	−0.02	0.04
Meghalaya	1.7	2.7	3.3	3.4	4.1	0.42	0.10	0.04
Mizoram	0.3	0.9	1.4	2.1	2.1	0.16	0.08	0.04
Nagaland	0.8	1.2	1.4	1.3	1.3	0.62	0.02	0.05
Delhi	0.0	0.5	0.5	1.7	1.4	0.00	−0.09	0.06
Odisha	1.7	2.6	3.0	3.5	3.4	0.50	0.13	0.02
Puducherry	0.0	2.1	1.9	2.8	2.1	0.00	−0.02	0.06
Punjab	2.6	2.3	1.1	1.4	1.2	2.18	−0.03	0.04
Rajasthan	0.7	1.0	1.0	1.0	0.9	0.75	0.02	0.03
Sikkim	0.0	1.4	1.2	0.9	0.7	0.00	−0.06	0.09
Tamil Nadu	4.3	4.3	3.7	3.3	3.4	1.26	−0.02	0.02
Tripura	2.6	4.5	2.8	2.4	2.7	0.97	−0.05	0.05
Uttar Pradesh	1.3	1.3	1.0	1.0	1.1	1.20	−0.06	0.02
Uttarakhand	1.6	1.0	0.7	1.0	1.1	1.41	0.03	0.05
West Bengal	3.5	3.0	3.4	4.0	2.2	1.54	−0.02	0.03
Telangana	2.0	3.0	3.8	3.8	3.6	0.55	0.06	0.04
**India**	**1.7**	**1.9**	**2.0**	**2.2**	**1.9**	**0.93**	**0.02**	**0.01**

As part of the spatial analysis, the first measure used in this study is global Moran’s I, which gives an indication of overall spatial autocorrelation in the dataset. The second measure is a LISA technique of local Moran’s I, which indicates the “presence or absence of significant spatial clusters or outliers for each location” in the dataset. The univariate and bivariate Moran’s I for Asthma and independent variables included in the analysis are presented in Table 4. The univariate Moran’s I value for Asthma is 0.47. It indicates high spatial auto-correlation in Asthma across the districts of India. Spatial autocorrelation is positive when similar values occur near one another in space. Results indicate that the districts with similar Asthma prevalence are near one another. The Moran’s I value for other independent variables are also significant; 0.45 for drinking water, 0.41 for clean cooking fuel, 0.44 for a household with no crowding, 0.45 for the common household environment and 0.43 for rural dwelling. It signifies that the Asthma and independent variables are correlated spatially across different districts of India.

**Table 4:** Moran’s I statistics showing spatial dependence of Asthma by different household environment and background characteristics across districts of India, 2015–16

Indicators	Univariate	Bivariate
% Asthma	0.48	**–**
% Tobacco Consumption	0.05	0.11
% Clean Cooking fuel	0.35	0.41
% Improved source of drinking water	0.42	0.45
% Household with no crowding	0.41	0.44
% Urban residence	0.33	0.38
% Rural residence	0.40	0.43
% Low household environment	0.27	0.33
% Medium household environment	0.42	0.45
% High household environment	0.22	0.31

### Explanation of Bivariate LISA maps

High clustering between Asthma and tobacco consumption, is observed in 7.00% districts out of 640 districts (**Map A**) which are also explained through the value of Moran’s I (0.11) whereas double the figure (14%) of the districts are having low clustering of Asthma and tobacco consumption. The districts identified in this map are Nadia, North 24 Pargana, South 24 Pargana, Kendujur, Anukul, Puri, Khordha, Dhenkanal, Jajapur, Kendrapara, Khammam, Warangal, Karimnagar, Medak, Hyderabad Rangareddy, East Godavari including Salem, Tiruvannamalai, Kanchipuram, Ariyarlur, Nagapattinam, Karaikal, Thiruvvarul, Pudukottai and Ramanathapuram from the southern regions. With a Moran’s I value of 0.41, **Map B** shows the clustering of districts for Asthma and cooking fuel depicting that 72 districts emerge to be the hotspots whereas 90 districts are identified as cold spots for Asthma with cooking fuel. The districts identified in the southern regions are Ernakulam, Malappuram, Coimbatore, Idukki, Potaiyyam, Kollam, Thiruvananthapuram, Kanyakumari, Tirunelveli, Thoothukuddi, Virudunagar, Ramanathapuram, Shiv Ganga, Madurai, Theni, Dindigul, Pudukottai, etc.. Another hotspot seen in the south consisted of districts like Prakasham, Guntur, Krishna, West Godavari, East Godavari, Nalgonda, Khammam, Warangal, Karimnagar, Medak, Rangareddy, Hyderabad and Chandrapur including districts from Jagatsinghpur to North 24 Pargana on the Eastern-coastal line. Similarly, **Map C** shows the spatial auto-correlation between Asthma with drinking water wherein about 11.00% of the districts have emerged as hotspot regions, while 99 districts have been the cold spot regions for drinking water. The Moran’s I value for drinking water, and Asthma is 0.45, which is reflected spatial correlation through Bi-LISA maps.

**Map D** shows the spatial correlation between Asthma and household with no crowding with a value of 0.44. The study finds that 99 districts are spatially correlated about Asthma and household with no crowding. The districts identified in the southern regions are Ernakulam, Malappuram, Coimbatore, Idukki, Potaiyyam, Kollam, Thiruvananthapuram, Kanyakumari, Tirunelveli, Thoothukuddi, Virudunagar, Ramanathapuram, Shiv Ganga, Madurai, Theni, Dindigul, Pudukottai, etc. Another major hotspot seen in the southern region itself consisted of districts like Prakasham, Guntur, Krishna, West Godavari, East Godavari, Nalgonda, Khammam, Warangal, Karimnagar, Medak, Rangareddy, Hyderabad, and Chandrapur including districts from Jagatsinghpur to North 24 Pargana on the Eastern-coastal line. It is clear from **Map E** that 67 districts are highly correlated with Asthma and urban residence supported by the value of Moran’s I (0.38) whereas 90 districts are having low autocorrelation. The clustering is found among the districts similar in Map B including West Garo Hills and West Tripura. **Map F**, which is for Asthma and rural residence; shows that about 11.00% of the districts are the hotspots regions which reflect the clustering through value of Moran’s I also (0.43). Similar kind of hotspots has been identified in the southern region whereas Sitamarhi, Muzaffarpur, Seohar, Purba Champaran and Chappra constitute the clustering in the northern districts except Chandrapur.

**Fig. 1:**
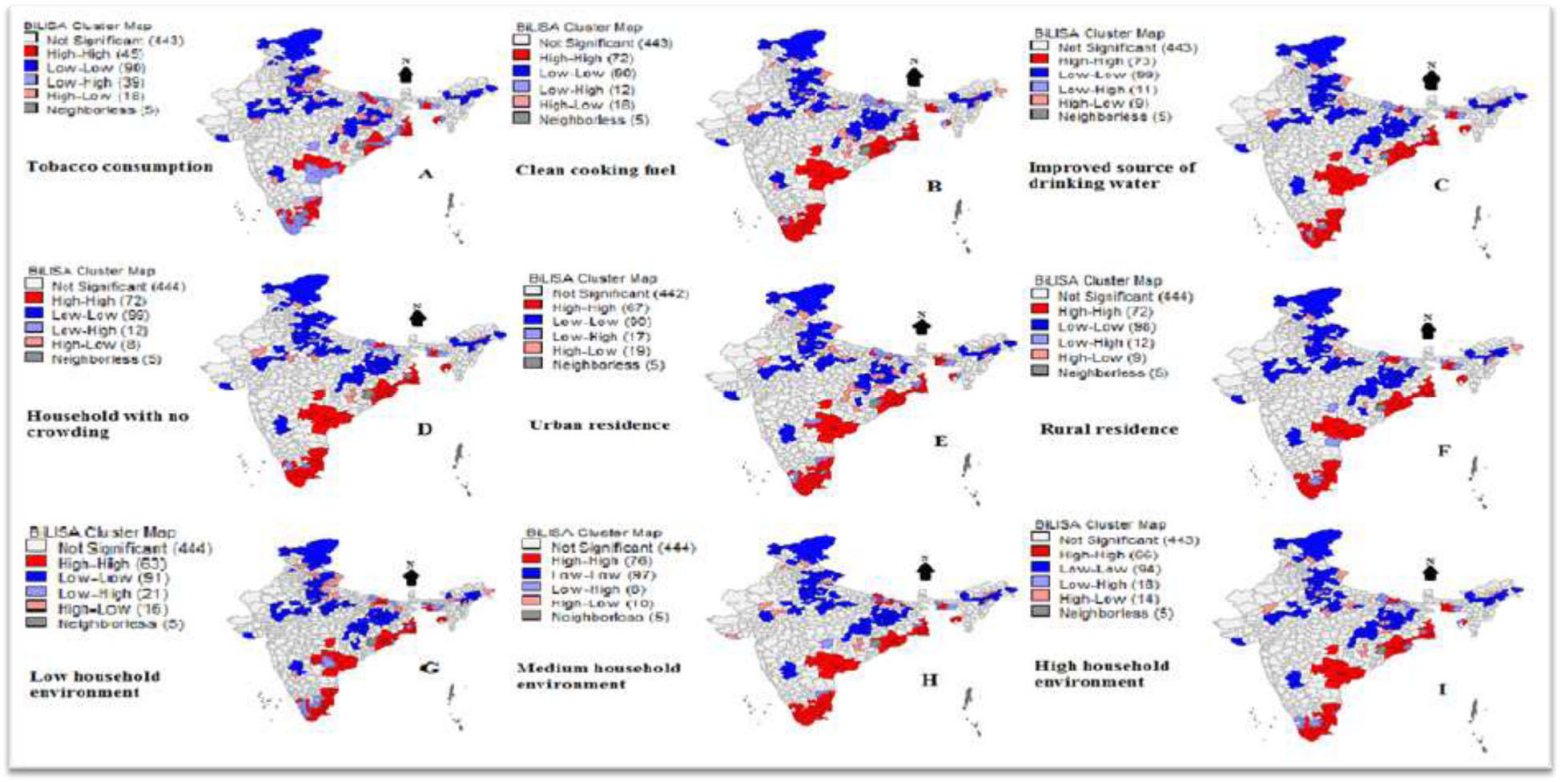
Bivariate LISA maps for Asthma and independent variables. Note: These maps are only indicative and do not portray the political/administrative boundaries of India. Some of the districts of Jammu and Kashmir have no data coverage [20]

The Moran’s I value for **Map G** is 0.33, which represent that 63 districts are having a high correlation between Asthma and low household environment. Comparatively, 91 districts were showing low clustering of Asthma with low household environments. It shows a similar pattern of clustering as Map F including Chandrapur. **Map H** shows that about 12.00% of the total districts are hotspots for Asthma (0.45) and medium household environment whereas 97 districts are having a low clustering of Asthma and medium household environment. Similar clustering has been found in the southern regions excluding Chandrapur and Purba Champaran. Finally, **Map I** depicts that 64 districts are having a high spatial correlation between Asthma and high household environment with Moran’s I value of 0.31. Around 94 districts are coming up as cold spots, and the pattern is almost same as previous one including Chandrapur, North Purba Champaran, and Sitamarhi (Fig. 1).

## Discussion

In the existing epidemiological transition in India, especially after the economic liberalization and development-oriented changes in the lifestyle of people, changing level of urbanization, economic prosperity, environmental and ecological factors have been considered as the major correlates of increasing prevalence of Asthma. However, the pattern of development and lifestyle changes including hygiene and sanitation have not been uniform across the country and vary to a large extent by caste, class, and social groups and resulting in differential prevalence in Asthma, chronic bronchitis and other risk factors for lung disorders. Therefore, the analysis in this paper has been organized by focusing at the meso- scale correlates as well as spatial heterogeneity in self-reported prevalence of Asthma among women aged 15–49 years across 640 districts in India using a nationally representative sample of 699,686 women from all 36 States/UTs.

It is worth mentioning that the prevalence of any NCD in a population-based survey based on the self-reported prevalence of the disease may be different from those estimated in a clinical setting, where the disease is diagnosed based on history, physical examination, and physiological testing. However, the estimated prevalence of Asthma of 1.94% among women aged 15–49 years seems quite reasonable in view of population-based prevalence of Asthma of 2.38% (2.21% among men and 2.56% among women) derived from data collected as part of a multicentre study from four cities namely Chandigarh, Delhi, Kanpur and Bangalore [21]. It is also coherent with the range of the current prevalence of Asthma from 1.20 to 6.30% among adults in most countries of the world [22, 23].

Results of the study highlight that women who consume tobacco in any form were significantly more likely to suffer from Asthma, which remains unchanged even after adjusting for some other predictors included in the model. These findings are more severe in the context of growing evidence of increasing prevalence of tobacco use among women in India [24]. This finding is similar to the results of other studies conducted in different parts of India. A study highlighted that the socio-economic gradient in NCDs and related risk factor distributions among population groups are also changing with increasing level of urbanization and growing proportion of slum population in India [25]. Tobacco consumption is now universally more common among the lower socio-economic groups and enhances their vulnerability to NCDs including Asthma, bronchitis and other forms of lung disorders. Another study conducted in slums of Pune, India have also concluded that along with other factors, smoking history was a significant risk factor associated with Asthma among the slum dwellers [26]. The interesting study conducted in different cities of India using a cross-sectional survey of more than 8000 subjects aged 23 years and above have resulted into similar findings and concluded that active smoking was observed to have an association with bronchial Asthma [21]. Another prospective, community-based study on school children also reported that active smoking was an independent risk factor for the development of Asthma like symptoms [27, 28]. In contrast, some other investigators have failed to demonstrate a significant association between tobacco smoking and Asthma in a variety of context [29, 30]. However, our study based on a nationally represented sample of women aged 15–49 years, traversing various socio-economic conditions and living in a range of environmental and ecological conditions affirm the relationship between tobacco use and prevalence of Asthma emerging from self-reported data collected as part of the national level household survey.

Another equally important issue that has emerged from this study is the higher prevalence of Asthma among women from the households who did not use a separate room as the kitchen. Because of the aforementioned situation, they were exposed to unclean fuels used for cooking, the impact of the fuels on Asthma patients quite similar to the effects of tobacco smoke on smokers. One approximate explanation to such findings may be that exposures of women to unclean fuel using in cooking may trigger recurrent induction of immune processes in lungs of women, which could compromise lung growth during developmental stages and making them susceptible to Asthma and chronic bronchitis [31, 32]. Women’s education has emerged as an important marker to the prevalence of Asthma as it was negatively associated with their educational attainment, i.e., higher the level of education of women, lesser was their likelihood of having Asthma. The intensity of the relationship was stronger with increasing level of schooling. The women who completed higher secondary level of educational attainment, were at a significantly lower risk of suffering from Asthma as compared to the women who did not have any schooling. Having a separate kitchen for cooking and women’s educational attainments are likely to be influenced by the economic development of households to which the study subjects belong. Therefore, any effort to curb the prevalence of Asthma may hinge around poverty and lifestyle-based classification of subjects, irrespective of urban-rural place of their residence.

The poor-rich ratios were higher in the economically progressive states like Kerala, Tamil Nadu, Maharashtra, Punjab, and economically backward states like Bihar, Uttar Pradesh, and West Bengal. Economically backward population from the hilly states of Himachal Pradesh, Uttarakhand and Jammu and Kashmir were in disadvantageous position with respect to prevalence of Asthma, as the PRRs were higher even in the hilly states, which have relatively larger exposure to cold weather and population in question might not be capable of adopting adequate protective measures.

## Conclusions

It is evident from the study that though the overall poor-rich ratio in the prevalence in Asthma at the national level does not portray profound variation by wealth quintiles, there is tremendous variability across States/UTs. Hence, there exists a considerable spatial variation by environmental and ecological factors. The prevelence of Asthma was found much above the national average for almost one-third of the districts in India. There is an evidence of better reporting of Asthma in certain group of women, especially those coming from economically better-off households and having better media exposure. Therefore, poverty-based variation in the prevalence of self-report morbidity should be analyzed with caution for any programme and interventions.

An analysis of spatial clustering in the prevalence of Asthma based on spatial autocorrelation portrays that the Moran’s I values were significant for an improved source of drinking water, clean fuel used for cooking, and household environment. The spatial dependence of Asthma on various background and behavioural characteristics of women indicate significantly higher spatial clustering in the prevalence of Asthma. Type of fuel used in cooking, women’s educational attainment and wealth quintiles of the households are some of the key predictors significantly explaining the spatial dependence of Asthma. These findings signify need to prioritize spatial clustering and neighbourhood characteristics while planning various public health intervention for the health and well-being of women in the reproductive ages across different districts of India. Some of the existing interventions in India are namely, Ujjala Yojana to ensure clean fuel for cooking, Swatch Bharat Mission enhancing awareness and practices to improved hygiene and sanitation and MPOWER strategies to strengthen the tobacco control program, which in turn may be useful in effectively addressing prevalence of Asthma and bronchitis among women in India and minimizing the risk factors for lung disorders. Efforts to curb the prevalence of Asthma through vertical interventions may hinge around the use of clean fuel, poverty, and lifestyle of subjects, irrespective of urban-rural place of their residence, environmental and ecological factors.

### Limitations of the study

The estimated prevalence of Asthma analysed in this study is based on self-reported cases among women aged 15–49 years interviewed as part of NFHS-4 (2015–16) and, hence, these estimates may only be treated as a proxy to total adult population-based burden of Asthma in India. There are various clinical evidence suggesting a relatively larger prevalence of Asthma among children and elderly population, and, hence, the burden of Asthma analysed in this study, may be somewhat lower than the true burden of Asthma in India.

## Data Availability

The data is available online for the public use at International Institute for Population Sciences (IIPS), Mumbai website. IIPS was the nodal agency for NFHS-4 survey; therefore, IIPS has data centre to make the availability of data. As being the faculty and student of this institute, we have accessed the data easily from institute’s data centre.

## Abbreviations

Bi-LISA: Bivariate Local Indicators of Spatial Association
CI: Concentration Index
DALYs: Disability-Adjusted Life Years
ECRHS: European Communities Respiratory Health Survey
GBD: Global Burden of Disease
GIS: Geographic Information System
IIPS: International Institute for Population Sciences
MoHFW: Ministry of Health and Family Welfare
NCDs: Non-communicable Disease
NHANES: National Health and Nutrition Examination Survey
PRR: Poor-Rich Ratios
UTs: Union Territories
WHO: World Health Organization
